# The Impact of Relocating a Trauma Center: Observations on Patient Injury Demographics and Resident Volumes

**DOI:** 10.7759/cureus.30256

**Published:** 2022-10-13

**Authors:** Viren Punja, Thomas Capasso, Kelley M Ray, Laura Stokes, Joel Narveson, Fang Niu, Neil D Patel, Kaily Ewing, Carlos A Fernandez, Jessica Veatch, Eirc Kuncir

**Affiliations:** 1 Trauma Surgery and Critical Care, Creighton University Medical Center, Omaha, USA; 2 Medicine, Creighton University School of Medicine, Omaha, USA; 3 Division of Clinical Research and Evaluate Sciences, Creighton University School of Medicine, Omaha, USA; 4 Trauma Surgery, Critical Care, and Acute Care Surgery, CHI Health Creighton University Bergan Mercy Medical Center, Omaha, USA; 5 Surgery, Creighton University School of Medicine, Omaha, USA

**Keywords:** resident education, geographic location, general surgery, medical education, trauma center

## Abstract

Introduction

Changing the physical zip code location of an academic trauma center may affect the distribution and surgical volume of its trauma patients. General surgical residency case log requirements may also be affected. This study describes the impact of moving a level I trauma center to a different zip code location, on the hospital and resident trauma case volumes.

Methods

This retrospective analysis included all patients within the local trauma registry across two fiscal years representing the pre- and post-move timeframes. Variables collected included patient basic sociodemographic and injury information, trauma activation level and transfer status, management (operative management [OPM] versus non-operative management [NOPM]), and resident case logs.

Results

During fiscal years 2016-2017 and 2017-2018, 3,025 patients were included. Pre-move and post-move trauma volumes were 1,208 and 1,817 respectively. Post-move changes demonstrated differences in basic sociodemographics, with differences in age (six years older), a shift toward white and away from black (12.89%), and males being seen more frequently (11.87%). Injury severity score distribution shifted (7.72%) towards less severe trauma scores (<15), the percentage of patients with blunt trauma (4.19%) and falls increased (ground level and greater than 1 meter, 9.78%) while the number of patients considered full activations were decreased (15.67%). Proportions of OPM and NOPM trauma cases remained unchanged with the exception of a reduction in emergent operative trauma (3.1%). Resident case logs requirements were met both pre- and post-move.

Conclusion

Relocating the trauma center to a different zip code location did not negatively impact our resident case volumes. Total trauma volumes were increased, with a shift in the demographics and severity distribution of injuries.

## Introduction

General surgical residency programs focus on developing the necessary skills for residents to practice in various settings after graduating. In specialties like trauma, taking calls confidently upon graduation necessitates extensive operative exposure. A caseload is critical for resident operative exposure and depends upon multiple factors. Hospital location can influence caseload based on sociodemographics, which are commonly disproportionately distributed within a city and can create geographic clustering of specific injury mechanisms [1,2]. For example, mechanisms such as gunshot wounds have the highest per capita incidence in resource-deprived counties, and patients tend to be non-white males, under the age of 40, and involved in an assault [1,2]. Unfortunately, patient outcomes also suffer disparities based on race, as white patients tend to have lower mortality and morbidity following a traumatic event [3-5]. Trauma centers tend to be placed in geographic locations based on the community's needs, which can also influence resident caseload.

Residents obtaining an adequate caseload to develop the necessary surgical skills has become more difficult with more trauma patients being managed nonoperatively [6-13]. Residents also spend substantial time performing non-educational hospital activities, which negatively impacts their surgical case volume and quality of life [14-16]. Moreover, the Accreditation Council for Graduate Medical Education (ACGME) implemented work hour restrictions leading to a night float system in order to reduce burnout [17,18] and, in 2018, the major operative case volume requirements were increased from 750 to 850 [19]. Reducing work hours and simultaneously increasing operative exposure may be challenging goals concurrently, especially with the current trends toward non-operative management (NOPM). These recent changes may unintentionally create barriers to developing the necessary surgical skills and intraoperative decision-making needed by surgery residents.

During the general time frame of ACGME changing case volume, the trauma center and residency program were physically moved from a more urban region with higher crime rates to a more suburban location. Ensuring residents have adequate exposure to meet the new ACGME surgical case volume requirements was of specific interest. The impact of this move on residency volumes and caseload demographics is unknown as no existing literature was found on this topic. The hypothesis was that moving the trauma center impacted the incidence of different mechanisms of injury in trauma patients, due to a variety of factors (geographic and sociodemographic, and socioeconomic factors), which will have influenced residents’ operative case volume.

## Materials and methods

The local Institutional Review Board approved this study as exempt research (#2002197), The Strengthening the Reporting of Observational Studies in Epidemiology Guidelines was reported (See Table 5 in the Appendix).

Study population

A retrospective review was conducted for patients who presented to the level one trauma center with traumatic injuries from July 1, 2016 to June 30, 2017 before the move and July 1, 2017 to June 30, 2018 after the move. These dates were chosen to allow comparison of relevant data between two locations pre- and post-move based upon academic years. All patients who were in the local trauma registry were included. All data points were retrieved from the local trauma registry and electronic medical records.

Study variables

Patient information including basic demographics (age, sex, and race) and injury demographics (Injury Severity Score [ISS], trauma type [blunt and penetrating], mechanism of injury [MOI]), level of trauma activation, and transfer-in status was collected.

Management data was collected including operative (OPM) versus non-operative (NOPM), service performing the OPM (trauma service or another service), if cases were emergent (< 24 hours or > 24 hours), and count of interventional radiology procedures.

The resident case logs were compared for the two classes who completed their trauma rotation within the selected timeframe. The logged number of cases of operative and non-operative trauma, and their role in procedures (surgeon chief, surgeon junior, teaching assistant, and first assistant) per class were compared.

Statistical analysis

All descriptive statistics were stratified pre- and post-move. Depending on data distribution, continuous variables were presented as mean ± SD or median and interquartile range, compared using the independent-samples t-test or Mann-Whitney test, respectively. Categorical variables were presented as frequency count and percent and compared using the chi-square test or Fisher's exact test. All analyses were conducted using SAS v. 9.4 with two-tailed p < 0.05 used to indicate statistical significance.

## Results

Volume and demographics

A total of 3,025 patients met inclusion criteria, with 1,208 (39.9%) patients pre-move and 1,817 (60.1%) post-move. Age, sex, race, and ethnicity differences were found to be statistically significant (Table 1). Post-move patients were an average of 6 years older, with an 11.4% shift from males to females, and race shifted towards seeing more White patients and fewer Black patients.

**Table 1:** Demographic characteristics for pre- and post-move Note. Data presented as mean ± SD or count (percent).

	Pre-move (n = 1208)	Post-move (n = 1817)	P
Age	46 (±21)	52 (±24)	<0.001
Sex			
Female	380 (31.46)	779 (42.87)	<0.001
Male	828 (68.54)	1038 (57.13)	
Race/ethnicity			
White	799 (66.14)	1436 (79.03)	<0.001
Black	262 (21.69)	157 (8.64)	
Hispanic	88 (7.28)	113 (6.22)	
Other	59 (4.88)	111 (6.11)	

Injury characteristics, transfer-in status, and activation levels

Median ISS were similar, however, there were statistical differences in the distribution of ISS scores (Table 2). Post-move there was a 4.6% increase in patients with ISS 0-9, 3.1% increase for ISS 10-14, while ISS 15-24 and >25 decreased by 3.3% and 4.4%, respectively. The type of trauma shifted towards blunt by 4.2% post-move which was statistically significant. Statistically and non-statistically significant differences were observed for various categories of MOI. Motor vehicle collisions (MVC), motorcycle collisions (MCC), and pedestrian/bicyclist were not different. In contrast, there were statistical differences noted in the victims of assaults by a 7.1% decrease, ground-level falls increased by 6.2%, falls greater than one meter increased by 3.6%, and a decrease in gunshot wounds (GSW) and stabbings by 1.5% and 1.2% respectively. There were statistical differences found for transfer-in status, with a reduction of patients transferred in by 4.7% post-move. Activation levels were statistically significant across all four categories; specifically, there was a 15.7% decrease in full activations and a 5.2% increase in partial activations, whereas consults increased by 3.2% and no activations by 7.3% (i.e., the patient walked in, was transferred in, or trauma service was consulted by the primary care team) after the move.

**Table 2:** Injury characteristics, transfer-in status and activation level for pre- and post-move Note. Data presented as median (IQR) or count (percent). Abbreviations: ISS = injury severity score; MVC = motor vehicle collision; MCC = motorcycle collision; and GSW = gunshot wound.

	Pre-move (n = 1208)	Post-move (n = 1817)	p
ISS	5 [3,10]	5 [3,10]	0.395
0-9	848 (70.20)	1359 (74.79)	0.005
10-14	137 (11.34)	263 (14.47)	0.013
15-24	112 (9.27)	108 (5.94)	<0.001
≥25	111 (9.19)	87 (4.79)	<0.001
Trauma Type			
Blunt	1057 (87.50)	1666 (91.69)	<0.001
Penetrating	151 (12.50)	151 (8.31)	<0.001
MOI			
MVC	305 (25.25)	516 (28.41)	0.056
Assault	151 (12.50)	98 (5.40)	<0.001
MCC	47 (3.89)	74 (4.07)	0.803
Ground Level Fall (Under 1m)	227 (18.79)	453 (24.94)	<0.001
Fall from Height (Over 1m)	195 (16.14)	359 (19.77)	0.012
Other Blunt	94 (7.78)	120 (6.61)	0.216
GSW	55 (4.55)	56 (3.08)	0.035
Stabbing	25 (2.07)	16 (0.88)	0.006
Other Penetrating	52 (4.30)	59 (3.25)	0.130
Pedestrian/Bicyclist Struck	57 (4.72)	65 (3.58)	0.118
Transfer in			
Yes	307 (25.41)	376 (20.69)	0.002
No	901 (74.59)	1441 (79.31)	0.002
Activation Level			
Full	399 (33.06)	316 (17.39)	<0.001
Partial	452 (37.45)	774 (42.60)	0.005
Consult	196 (16.24)	353 (19.43)	0.025
None	160 (13.26)	374 (20.58)	<0.001

OPM versus NOPM

There were no statistically significant differences observed for OPM post-move for any variables except for a 3.1% reduction in operative traumas performed in <24 hours (Table 3). NOPM was different in the proportions of blunt versus penetrating trauma after the move, with a 4.4% increase in blunt and a 3.0% reduction in penetrating. Types of trauma procedures performed remained relatively unchanged with the exception of neck exploration occurring less post-move. The utilization of interventional radiology procedures was not changed after the move.

**Table 3:** Operative and non-operative management for pre- and post-move Note. Data presented as count (percent).

	Pre-move (n = 1208)	Post-move (n = 1817)	p
Operative			
Yes	346 (28.64)	495 (27.24)	0.400
Mechanism			
Blunt	287 (23.76)	428 (23.56)	0.896
Penetrating	59 (4.88)	67 (3.68)	0.106
Operative By Trauma	120 (9.94)	109 (5.97)	<0.001
<24 Hours	82 (6.79)	67 (3.69)	<0.001
>24 Hours	38 (3.15)	42 (2.31)	0.161
No	862 (71.36)	1322 (72.76)	0.400
Mechanism			
Blunt	770 (63.74)	1238 (68.13)	0.012
Penetrating	92 (7.62)	84 (4.63)	<0.001
Types of Trauma Procedures Performed	151 (12.50)	245 (13.48)	0.432
Bowel Resection	0	6 (0.33)	0.113
Exploratory Laparotomy	31 (2.57)	70 (3.85)	0.053
Fasciotomy	4 (0.33)	7 (0.39)	0.949
Neck Exploration	14 (1.16)	9 (0.50)	0.039
Rib Fixation	6 (0.50)	7 (0.39)	0.645
Splenectomy	4 (0.33)	4 (0.22)	0.824
Thoracotomy	13 (1.08)	20 (1.10)	0.949
Tracheostomy and Percutaneous Endoscopic Gastrostomy	9 (0.75)	11 (0.61)	0.642
Video Assisted Thoracoscopic Surgery and Cryoablation	16 (1.32)	12 (0.66)	0.061
Wound Exploration	54 (4.47)	99 (5.45)	0.229
Count of Interventional Radiology Procedures			
Yes	31 (2.56)	51 (2.81)	0.862
No	1117 (97.4)	1766 (97.2)	0.862

Resident case logs

Eight resident case logs were analyzed during the time period of the study. The ACGME resident case logs revealed comparable numbers of total major cases per chief resident pre- and post-move (Table 4). The only difference between the years was a more than two-fold increase in non-operative and surgical critical care cases. See Table 6 in the Appendix for more details on resident surgical cases.

**Table 4:** Resident Case Logs - Median cases Note. Values are median and (range)

	Pre-move	Post-move	p
Total Major Cases	1100 (960-1337)	1103 (898-1283)	0.882
Operative Trauma	29 (20-34)	25 (19-26)	0.294
Non-Operative Trauma	25 (20-41)	56 [55-97)	0.025
Surgical Critical Care	20 (15-27)	43 (41-44)	0.025
Surgeon Chief	214 (187-292)	278 (202-288)	0.655
Teaching Assist	38 (30-55)	43 (30-54)	0.430

## Discussion

Maintaining a successful residency program requires ongoing restructuring, in response to changes in medicine and ACGME standards, to best prepare residents to function independently upon graduation. There has been a paradigm shift in trauma towards greater utilization of non-operative interventions, aided by improved high-resolution imaging and advances in interventional radiology [9,10]. Sociodemographics, race, geography, and mechanism of injury are all interrelated factors that influence resident caseload and ultimately operative experience gained during residency. No prior evidence has investigated the impact relocating a trauma center might have on resident caseload and operative exposure. The relocation of the level-one trauma center influenced the patient demographics, some of the injury patterns, and the proportion of OPM versus NOPM while maintaining the resident’s total operative volumes.

Trauma and surgical volume

Trauma volume increased by 609 and operative volume by 149 after the move and thus the change in trauma center location may have helped surgical residents receive adequate trauma experience. Although overall trauma volume increased, the percentage of operative volume decreased by 1.4%. Many technological changes have occurred in recent years that have impacted surgical volumes; for example, marked improvements in computed tomography (CT) scans and IR have reduced the need for surgical exploration [6-13]. Currently, 80% of trauma cases are managed nonoperatively with penetrating trauma requiring emergent surgery more frequently than blunt injuries [13]. A higher resident caseload may help maintain surgical case volumes despite NOPM trends.

Patient demographics

There were statistical differences noted in all basic demographic variables collected. Age was different by six years; older patients tend to have more complications, longer hospital stays, increased costs, and worse functional outcomes [20,21], and their mechanism of injury differ from younger patients [22]. However, the clinical significance of this minor age difference is questionable given that both groups were middle-aged without crossing into extremes of the age range. Thus, the impact of age on resident surgical exposure or case volumes is questionable. Sex and race were also statistically different, with more males (11.87%) and White patients (12.89%) after the move. Males tend to have greater associations with penetrating trauma than females, and white patients are less often associated with assault and gunshot wounds [1,2], so the change in sex and race distribution may have contributed to alterations in injury patterns observed.

Injury characteristics, transfer-in status, and activation levels

After moving hospital locations, there was no change in median ISS, however, the groups’ ISS distributions differed. An increase was observed in patients with ISS < 15 and a decrease in those with ISS ≥ 15 post-move. These differences were statistically significant and clinically important. ISS scores ≥ 15 are considered major trauma [20] and higher ISS are associated with greater mortality risk [23]. Consequential to the reduction in the severity of trauma post-move, resident experience with high complexity cases may be reduced. Simultaneously, more blunt trauma was seen which may explain why the MOI was seen to involve more falls, fewer assaults, gunshot wounds, and stabbings. Blunt traumas are more likely to receive non-operative care, as compared to penetrating traumas [7,8]. The increase in the blunt trauma activations post-move may also be partly influenced by the proximity to a major interstate highway, due to MVCs post-move with a clinically meaningful trend. Additionally, there were fewer full activations (15.67%), while all other activations categories were increased. This correlates with the decreases in ISS and penetrating trauma seen since the move. Thus, the changes in injury type, severity, MOI, transfer status, and activation level seemed to move together in a predictable fashion given the new location of the trauma center was in an area with an older population with a higher socioeconomic status. The aforementioned changes may afford residents a greater opportunity for critical care experience, albeit with lower acuity, while also reducing their operative exposure.

OPM versus NOPM

The only statistically significant difference in OPM was performing fewer emergency operations (<24 hours) and NOPM noted more blunt types of trauma. This change may be due to differences in injury mechanisms observed at the new location. For example, falls from any height were more common post-move, which may predispose towards more orthopedic surgeries due to hip and extremity fractures, whereas GSW and stabbings both decreased, which would typically be emergent operations performed by the trauma service. An increase was observed in IR procedures that were not statistically significant. It is unlikely this change had any short-term impact on resident case volumes.

Resident case logs

The resident case logs demonstrated that total case volume was unchanged pre- and post-move, and residents were able to achieve the required number of cases per ACGME regardless of location. Types of trauma procedures performed were essentially unchanged, the differences observed in neck exploration might be explained by the shifts in blunt and penetrating trauma post-move. Operative volume is important as higher volumes are associated with enhanced proficiency and outcomes [14,24]. Qualitative surveys of residents who logged more than 950 cases report greater confidence in their skills and ability to take calls at a level one trauma center [25]. While there are concerns that work hour restrictions may negatively affect surgical volume, lead to non-compliance and falsification of timesheets, and the night float system could reduce operative volume [21,26]. Contrarily, some residents reported better quality of life [17,27] while other studies did not see an impact on case volumes or written boards pass rates [28]. Changes were not observed in case volume pre- or post-move, and total surgical volumes were maintained above the ACGME requirements. All logged cases, regardless of role, count towards the total case required for graduation, however, 200 of which must occur during the resident’s chief year [19,24,25]. Similarly, there was no difference between years for surgeon chief volumes and ACGME requirements continued to be exceeded.

The location change did bring a greater proportion of blunt trauma cases and therefore the increase in non-operative trauma cases and more surgical critical care experience is anticipated. Exposure to more critical care cases is positive, although it is important to recognize not all critical care cases afford equivalent experience and opportunities for clinical reasoning. Due to the lower ISS scores, the residents may not have actually seen more critically ill patients while also having less opportunity to develop surgical skills. Nevertheless, post-move, operative trauma was insignificantly decreased, suggesting that the two domains were balanced. Furthermore, the ACGME required operative (10 cases) and non-operative trauma volumes (40 cases, [29]) were exceeded by the residents in the program. Overall, volumes were maintained above the required operative caseload set by the ACGME and were generally comparable to national averages both pre and post-move [30]. This seemingly affirms that a sufficient caseload to acquire surgical expertise is provided by this residency, despite the work hour restrictions and unimpacted by the change in trauma center location.

Weaknesses and strengths

This study is principally limited by the retrospective nature of the design limiting the ability to analyze for cause and effect as well as not evaluating the resident's perceived readiness for practice at the time of graduation. Conclusions are further limited in generalizability due to only a single trauma center being studied. Although attempts were made to understand the data collected, there is no way to discern cause and effect. Sociodemographics, the presence of two trauma centers in the city, and EMS and other pre-hospital providers' idiosyncratic protocols all undoubtedly influenced the results of this study in a way that cannot be quantified or explained with the nature of this study. Operative case volumes are susceptible to numerous factors, for example, clinical decision-making, which was not measured in any capacity in this study and may confound the results. Furthermore, it was assumed residents counted all their cases and didn't stop counting once they met their requirements. One of the strengths of this study was looking at comparable timeframes of data, i.e., a year before and after the move. Long-term follow-up could be an interesting future study.

## Conclusions

This is the first study to examine the effects of moving a trauma center location on patient and resident surgical case volumes. Moving the trauma center to a new zip code location shifted the trauma patient demographics toward older, white males. The injuries observed post-move were less severe, with more blunt trauma and ground-level falls. OPM remained the same except for a reduction in emergent trauma. Resident case logs remained above ACGME requirements with a more than two-fold increase in non-operative and surgical critical care cases. The Relocation of a level-one trauma center shifted patient demographics while maintaining the residency program's case volume.

## Appendices

**Table 5:** STROBE Statement - checklist of items that should be included in reports of cross-sectional studies

	Item No	Recommendation	Section/Page Number
Title and abstract	1	(a) Indicate the study’s design with a commonly used term in the title or the abstract	Title Page, Abstract
		(b) Provide in the abstract an informative and balanced summary of what was done and what was found	Abstract
Introduction			
Background/rationale	2	Explain the scientific background and rationale for the investigation being reported	Background
Objectives	3	State specific objectives, including any prespecified hypotheses	Background
Methods			
Study design	4	Present key elements of study design early in the paper	Methods “study population”
Setting	5	Describe the setting, locations, and relevant dates, including periods of recruitment, exposure, follow-up, and data collection	Methods “study population”
Participants	6	(a) Give the eligibility criteria, and the sources and methods of selection of participants	Methods “study population”
Variables	7	Clearly define all outcomes, exposures, predictors, potential confounders, and effect modifiers. Give diagnostic criteria, if applicable	Methods “Study variables”
Data sources/ measurement	8*	For each variable of interest, give sources of data and details of methods of assessment (measurement). Describe comparability of assessment methods if there is more than one group	Methods “study population” and “Study variables”
Bias	9	Describe any efforts to address potential sources of bias	“Study variables”
Study size	10	Explain how the study size was arrived at	“Statistical Analysis”
Quantitative variables	11	Explain how quantitative variables were handled in the analyses. If applicable, describe which groupings were chosen and why	“Statistical Analysis”
Statistical methods	12	(a) Describe all statistical methods, including those used to control for confounding	“Statistical Analysis”
		(b) Describe any methods used to examine subgroups and interactions	
		(c) Explain how missing data were addressed	
		(d) If applicable, describe analytical methods taking account of sampling strategy	
		(e) Describe any sensitivity analyses	
Results			
Participants	13*	(a) Report numbers of individuals at each stage of study—eg numbers potentially eligible, examined for eligibility, confirmed eligible, included in the study, completing follow-up, and analysed	“Results”
		(b) Give reasons for non-participation at each stage	
		(c) Consider use of a flow diagram	
Descriptive data	14*	(a) Give characteristics of study participants (eg demographic, clinical, social) and information on exposures and potential confounders	“Results”
		(b) Indicate number of participants with missing data for each variable of interest	
Outcome data	15*	Report numbers of outcome events or summary measures	“ Results”
Main results	16	(a) Give unadjusted estimates and, if applicable, confounder-adjusted estimates and their precision (eg, 95% confidence interval). Make clear which confounders were adjusted for and why they were included	“Results”
		(b) Report category boundaries when continuous variables were categorized	
		(c) If relevant, consider translating estimates of relative risk into absolute risk for a meaningful time period	
Other analyses	17	Report other analyses done—eg analyses of subgroups and interactions, and sensitivity analyses	
Discussion			
Key results	18	Summarise key results with reference to study objectives	“Discussion”
Limitations	19	Discuss limitations of the study, taking into account sources of potential bias or imprecision. Discuss both direction and magnitude of any potential bias	“Weaknesses and Strengths”
Interpretation	20	Give a cautious overall interpretation of results considering objectives, limitations, multiplicity of analyses, results from similar studies, and other relevant evidence	“Discussion”
Generalisability	21	Discuss the generalisability (external validity) of the study results	“Weaknesses and Strengths”
Other information			
Funding	22	Give the source of funding and the role of the funders for the present study and, if applicable, for the original study on which the present article is based	“Conflicts of interest and source of Funding”

**Table 6:** Resident case logs - case details and average number of cases per resident

Case Description	Pre-move Average	Post-move Average
Expl Thoracotomy-Thoracoscopic	0.00	0.33
Expl Thoracotomy-Open	2.25	2.33
Explor Laporotomy-Laproscopic	1.50	0.33
Expl Laporotomy-Open	6.20	5.67
Colon Trauma-Closure/Resect/Exclusion	1.50	0.67
Sm Bowel Trauma- Closure/Resect/Exclusion	1.50	0.67
Neck Explor for Trauma	1.67	1.33
Splenectomy/Splenorrhaphy-Open	1.00	2.33
Debride/Suture Major Wounds	2.00	1.33
Repair/Drainage Hepatic Lacs-Open	2.33	0.67
Repair/Drainage/Resection of Pancreatic Injury	1.00	0.33
Fasciotomy for injury	1.33	1.00
Repair Peripheral Vessels	1.50	0.33
Other Major Trauma	2.00	0.33

## References

[1] Abaza R, Lukens-Bull K, Bayouth L, Smotherman C, Tepas J, Crandall M (2020). Gunshot wound incidence as a persistent, tragic symptom of area deprivation. Surgery.

[2] Newgard CD, Schmicker RH, Sopko G (2011). Trauma in the neighborhood: a geospatial analysis and assessment of social determinants of major injury in North America. Am J Public Health.

[3] Mikhail JN, Nemeth LS, Mueller M, Pope C, NeSmith EG (2018). The social determinants of trauma: a trauma disparities scoping review and framework. J Trauma Nurs.

[4] Hsia RY, Shen YC (2011). Rising closures of hospital trauma centers disproportionately burden vulnerable populations. Health Aff (Millwood).

[5] Cook A, Harris R, Brown HE, Bedrick E (2020). Geospatial characteristics of non-motor vehicle and assault-related trauma events in greater Phoenix, Arizona. Inj Epidemiol.

[6] Fakhry SM, Watts DD, Michetti C, Hunt JP (2003). The resident experience on trauma: declining surgical opportunities and career incentives? Analysis of data from a large multi-institutional study. J Trauma.

[7] Lukan JK, Carrillo EH, Franklin GA, Spain DA, Miller FB, Richardson JD (2001). Impact of recent trends of noninvasive trauma evaluation and nonoperative management in surgical resident education. J Trauma.

[8] Bulinski P, Bachulis B, Naylor DF Jr, Kam D, Carey M, Dean RE (2003). The changing face of trauma management and its impact on surgical resident training. J Trauma.

[9] Drake FT, Aarabi S, Garland BT (2017). Accreditation Council for Graduate Medical Education (ACGME) surgery resident operative logs: the last quarter century. Ann Surg.

[10] McKenna DT, Mattar SG (2014). What is wrong with the training of general surgery?. Adv Surg.

[11] Hawkins ML, Wynn JJ, Schmacht DC, Medeiros RS, Gadacz TR (1998). Nonoperative management of liver and/or splenic injuries: effect on resident surgical experience. Am Surg.

[12] Jennings GR, Poole GV, Yates NL, Johnson RK, Brock M (2001). Has nonoperative management of solid visceral injuries adversely affected resident operative experience?. Am Surg.

[13] Watkins CJ, Feingold PL, Hashimoto B, Johnson LS, Dente CJ (2012). Nocturnal violence: implications for resident trauma operative experiences. Am Surg.

[14] Ferguson CM, Kellogg KC, Hutter MM, Warshaw AL (2005). Effect of work-hour reforms on operative case volume of surgical residents. Curr Surg.

[15] Landmann A, Mahnken H, Antonoff MB, White S, Patel A, Scifres AM, Lees JS (2017). Keeping residents in the dark: do night-float rotations provide a valuable educational experience?. J Surg Educ.

[16] Chung RS, Ahmed N (2007). How surgical residents spend their training time: the effect of a goal-oriented work style on efficiency and work satisfaction. Arch Surg.

[17] Christmas AB, Brintzenhoff RA, Sing RF, Schmelzer TM, Bolton SD, Miles WS, Thomason MH (2009). Resident work hour restrictions impact chief resident operative experience. Am Surg.

[18] de Virgilio C, Yaghoubian A, Lewis RJ, Stabile BE, Putnam BA (2006). The 80-hour resident workweek does not adversely affect patient outcomes or resident education. Curr Surg.

[19] (2022). Accreditation Council for Graduate Medical Education. Common program requirement. https://www.acgme.org/globalassets/PFAssets/ProgramRequirements/440_GeneralSurgery_2020.pdf.

[20] Pecheva M, Phillips M, Hull P, Carrothers A OR, Queally JM (2020). The impact of frailty in major trauma in older patients. Injury.

[21] MacKenzie EJ, Weir S, Rivara FP (2010). The value of trauma center care. J Trauma.

[22] Feero S, Hedges JR, Simmons E, Irwin L (1995). Intracity regional demographics of major trauma. Ann Emerg Med.

[23] Alberdi F, García I, Atutxa L, Zabarte M (2014). Epidemiology of severe trauma. Med Intensiva.

[24] Cortez AR, Katsaros GD, Dhar VK (2018). Narrowing of the surgical resident operative experience: a 27-year analysis of national ACGME case logs. Surgery.

[25] DuCoin C, Hahn A, Baimas-George M, Slakey DP, Korndorffer JR Jr (2018). The change in surgical case diversity over the past 15 years and the influence on the pursuit of surgical fellowship. Am Surg.

[26] Coverdill JE, Alseidi A, Borgstrom DC (2016). Professionalism in the twilight zone: a multicenter, mixed-methods study of shift transition dynamics in surgical residencies. Acad Med.

[27] Abraham T, Freitas M, Frangos S, Frankel HL, Rabinovici R (2006). Are resident work-hour limitations beneficial to the trauma profession?. Am Surg.

[28] Damadi A, Davis AT, Saxe A, Apelgren K (2007). ACGME duty-hour restrictions decrease resident operative volume: a 5-year comparison at an ACGME-accredited university general surgery residency. J Surg Educ.

[29] (2022). Accreditation Council for Graduate Medical Education. Minimum Numbers for General Surgery Residents and Credit Role. https://www.acgme.org/globalassets/definedcategoryminimumnumbersforgeneralsurgeryresidentsandcreditrole.pdf.

[30] (2022). Accreditation Council for Graduate Medical Education Surgery Case Logs National Data Report. https://apps.acgme-i.org/ads/Public/Reports/Report/25.

